# Supplementary material for: Genetic variation in a plant-parasitic nematode is associated with host gene expression changes conserved across evolutionarily distant host plants
Source: G3 (Bethesda). 2026 Jul 27;16(10):jkag204. doi: 10.1093/g3journal/jkag204 (PMC13643774; doi:10.1093/g3journal/jkag204)
Supplement: Supplementary file 1 [file jkag204_supplementary_data.zip › Supplemental_Figure_S1_G3-2025-406321.docx]

**Supplemental Figure S1.** Volcano plots displaying the results of the tests of differential expression for each plant host species (VW9-infected plants vs. LM-infected plants). The x-axis provides the log_2_ fold-change, and the y-axis is the -log_10_ p-value. Statistically significant results are indicated in blue.
