# Supplementary material for: Genetic variation in a plant-parasitic nematode is associated with host gene expression changes conserved across evolutionarily distant host plants
Source: G3 (Bethesda). 2026 Jul 27;16(10):jkag204. doi: 10.1093/g3journal/jkag204 (PMC13643774; doi:10.1093/g3journal/jkag204)
Supplement: Supplementary file 1 [file jkag204_supplementary_data.zip › Supplemental_Table_S1_G3-2025-406321.docx]

|  | **N genes up-expressed (VW9- vs LM-infected)** | | | | | | | | | | | | | | |
| --- | --- | --- | --- | --- | --- | --- | --- | --- | --- | --- | --- | --- | --- | --- | --- |
| **N significant genes in orthogroup** |  | **0** | **1** | **2** | **3** | **4** | **5** | **6** | **7** | **8** | **9** | **10** | **11** | **12** | **13** |
|  | **2** | 0 | 1 | 0 |  |  |  |  |  |  |  |  |  |  |  |
|  | **3** | 0 | 0.889 | 0.889 | 0 |  |  |  |  |  |  |  |  |  |  |
|  | **4** | 0 | 0.75 | 1 | 0.75 | 0 |  |  |  |  |  |  |  |  |  |
|  | **5** | 0 | 0.64 | 0.96 | 0.96 | 0.64 | 0 |  |  |  |  |  |  |  |  |
|  | **6** | 0 | 0.556 | 0.889 | 1 | 0.889 | 0.556 | 0 |  |  |  |  |  |  |  |
|  | **7** | 0 | 0.49 | 0.816 | 0.98 | 0.98 | 0.816 | 0.49 | 0 |  |  |  |  |  |  |
|  | **8** | 0 | 0.438 | 0.75 | 0.938 | 1 | 0.938 | 0.75 | 0.438 | 0 |  |  |  |  |  |
|  | **9** | 0 | 0.395 | 0.691 | 0.889 | 0.988 | 0.988 | 0.889 | 0.691 | 0.395 | 0 |  |  |  |  |
|  | **10** | 0 | 0.36 | 0.64 | 0.84 | 0.96 | 1 | 0.96 | 0.84 | 0.64 | 0.36 | 0 |  |  |  |
|  | **11** | 0 | 0.331 | 0.595 | 0.793 | 0.926 | 0.992 | 0.992 | 0.926 | 0.793 | 0.595 | 0.331 | 0 |  |  |
|  | **12** | 0 | 0.306 | 0.556 | 0.75 | 0.889 | 0.972 | 1 | 0.972 | 0.889 | 0.75 | 0.556 | 0.306 | 0 |  |
|  | **13** | 0 | 0.284 | 0.521 | 0.71 | 0.852 | 0.947 | 0.994 | 0.994 | 0.947 | 0.852 | 0.71 | 0.521 | 0.284 | 0 |
|  | **18** | 0 | 0.21 | 0.395 | 0.556 | 0.691 | 0.802 | 0.889 | 0.951 | 0.988 | 1 | 0.988 | 0.951 | 0.889 | 0.802 |

**Supplemental Table S1: Scaled variances of direction of effect (up- vs down-expressed genes) for each size of orthogroup.** Orthogroup size represents numbers of genes differentially expressed across the experiment (based on the counts from the real data). Scaled variance is calculated as $4\left( 1/N_{g} \right)\sum_{i} \left( x_{i}-\bar{x} \right)^{2}$, where *N_g_* is the number of significant genes in the orthogroup and *x_i_* is the indicator variable for each gene. This scaled variance will be 0 if the direction of expression change is the same for all the genes in the orthogroup. It will be 1 if the expression change for half the genes is in one direction and is in the opposite direction for the other half of the genes. For example, when two genes are differentially expressed in an orthogroup (the first row), and both of them are up-expressed (VW9- vs. LM-infected) or both are down-expressed (first and third columns, respectively), the scaled variance is 0. If one gene is up-expressed and the other down-expressed (second column), the scaled variance is 1. The table is truncated for the final row, as the values are symmetric around a scaled variance of 1.
